# Protective effects of low-magnitude high-frequency vibration on high glucose-induced osteoblast dysfunction and bone loss in diabetic rats

**DOI:** 10.1186/s13018-021-02803-w

**Published:** 2021-10-30

**Authors:** Zhaoyu Fu, Xu Huang, Pengcheng Zhou, Bo Wu, Long Cheng, Xinyu Wang, Dong Zhu

**Affiliations:** 1 Department of Orthopaedic Trauma, The First Hospital of Jilin University, Changchun, Jilin China; 2 Department of Radiology, The First Hospital of Jilin University, Changchun, Jilin China

**Keywords:** Low-magnitude high-frequency vibration, Osteoblast, High glucose, Diabetic rat, Bone loss

## Abstract

**Objective:**

Low-magnitude high-frequency vibration (LMHFV) has been reported to be capable of promoting osteoblast proliferation and differentiation. Reduced osteoblast activity and impaired bone formation were related to diabetic bone loss. We investigated the potential protective effects of LMHFV on high-glucose (HG)-induced osteoblasts in this study. In addition, the assessment of LMHFV treatment for bone loss attributed to diabetes was also performed in vivo.

**Method:**

MC3T3-E1 cells induced by HG only or treated with LMHFV were treated in vitro. The experiments performed in this study included the detection of cell proliferation, migration and differentiation, as well as protein expression. Diabetic bone loss induced by streptozotocin (STZ) in rats was established. Combined with bone morphometric, microstructure, biomechanical properties and matrix composition tests, the potential of LMHFV in treating diabetes bone loss was explored.

**Results:**

After the application of LMHFV, the inhibiting effects of HG on the proliferation, migration and differentiation of osteoblasts were alleviated. The GSK3β/β-catenin pathway was involved in the protective effect of LMHFV. Impaired microstructure and biomechanical properties attributed to diabetes were ameliorated by LMHFV treatment. The improvement of femur biomechanical properties might be associated with the alteration of the matrix composition by the LMHFV.

**Conclusion:**

LMHFV exhibited a protective effect on osteoblasts against HG by regulating the proliferation, migration and differentiation of osteoblasts. The function of promoting bone formation and reinforcing bone strength made it possible for LMHFV to alleviate diabetic bone loss.

**Supplementary Information:**

The online version contains supplementary material available at 10.1186/s13018-021-02803-w.

## Introduction

Osteoporosis includes primary and secondary osteoporosis. Primary osteoporosis is associated with menopause and old age, while secondary osteoporosis is generally attributed to diseases or drugs [1]. Diabetes refers to one of the most common diseases causing osteoporosis. Varying levels of bone loss have been reported in patients with type 1 and type 2 diabetes [2]. Moreover, increased risks of hip, spine and ankle fracture were also considered to be associated with the progression of diabetes [3]. The balance between bone formation and bone resorption is disrupted due to diabetes, with variations in bone microstructure and the occurrence of fragile bones [4]. Even antidiabetic drugs such as insulin and metformin were reported to exert side effects on bone and cause increased risk of fracture [5]. Although some alternative medicine methods, such as physiotherapy and dietetic therapy, have been employed for treating diabetes-induced osteoporosis, it remains a common and severe problem.

In a range of animal experiments performed since 2001, when mechanical vibrations were initially reported to increase the density of proximal femoral trabeculae in sheep [6], mechanical vibrations with low magnitude (0.3 g) and high frequency (20–60 Hz), termed LMHFVs, were confirmed to facilitate osteogenesis in vivo [7, 8]. Evidence from clinical trials also supported that LMHFV could increase bone density in postmenopausal women [9]. The application of LMHFV has been recently broadened with the deepening of its practice, and clinical effects have been identified in patients with obesity and neurological disorders [10, 11]. Improvements in muscle strength, body composition, balance, and quality of life were reported in diabetic patients who had undergone mechanical vibration treatments [12]. Thus, LMHFV might help mitigate bone loss induced by diabetes.

A previous study reported that LMHFV could improve osteocyte viability in a high-glucose environment [13]. However, for osteoblasts that are sensitive to mechanical stress and responsible for the formation of bone matrix and calcification, whether these cells can be improved from LMHFV under hyperglycaemic conditions remains unclear. The hypoglycaemic effect of LMHFV was identified in diabetic rats, while the effect of LMHFV on the structure and mechanical properties of bones has not been clarified [14]. Thus, the present study investigated the effects of LMHFV on the cell function of osteoblast cells induced high glucose in vitro and bone loss in a diabetic rat model in vivo.

## Methods

### Cell culture and reagents

MC3T3-E1 cells originated from Zhong Qiao Xin Zhou Biotechnology Co., Ltd. (Shanghai, China). Minimum essential medium-alpha modification (α-MEM) and foetal bovine serum (FES) were provided by Gibco (Gaithersburg, USA). MC3T3-E1 cells were cultured with α-MEM supplemented with 10% FBS at 37 °C in a 5% CO2 atmosphere.

High-glucose (25.0 mmol/L) medium was produced by adding extra D-glucose purchased from Sigma–Aldrich Co., Ltd. (Shanghai, China) to the ordinary medium. β-glycerophosphate and ascorbic acid were purchased from Aladdin Co., Ltd. (Shanghai, China), and β-glycerophosphate (10 mM) and ascorbic acid (50 mg/mL) dissolved in α-MEM supplemented with 10% FBS acted as the osteogenesis medium. Anti-GSK3β, anti-p-GSK3β (S9), anti-β-catenin, anti-Cyclin D1, anti-Cmyc, and anti-Runx2 were purchased from Abcam (Cambridge, UK). Alizarin Red S staining solution, propidium iodide solution and DAPI solution were purchased from Solarbio Co., Ltd. (Beijing, China). Furthermore, Beyotime Biotechnology (Suzhou, China) provided an EdU Cell Proliferation Kit with Alexa Fluor 555, BCIP/NBT Alkaline Phosphatase Colour Development Kit, Alkaline Phosphatase Assay Kit, anti-β-actin, HRP-labelled secondary antibody and Enhanced Chemiluminescence Kit. XAV939 was purchased from MedChemExpress Company (New Jersey, USA).

### Treatment with high glucose and the application of LMHFV

By convention, MC3T3-E1 cells with 25 mmol/L glucose contained in the medium acted as high-glucose (HG)-induced osteoblasts in vitro. LMHFV with a magnitude of 0.25 g, a frequency of 35 Hz and an interval of 15 min a day was applied to HG-induced MC3T3-E1 cells in vitro through the cell vibration platform, and the vibration was monitored with a vibration monitor. The cell vibration platform was designed by the research group of the authors [15], and the vibration monitor was purchased from Xingsheng Co., Ltd. (Shanghai, China). MC3T3-E1 cells were cultured normally with the medium changed regularly and acted as the normal contrast group (NC). MC3T3-E1 cells cultured with medium supplemented with 25 mmol/L glucose acted as the HG-induced group (HG). MC3T3-E1 cells induced by 25 mmol/L glucose and LMHFV acted as the LMHFV group (LMHFV).

### EdU assay

MC3T3-E1 cells were seeded into 6-well plates and then cultured with different treatments for 5 days. Subsequently, the medium was changed, and EdU was added at a final concentration of 10 μM. After incubation for 1 h and 30 min, the cells were fixed with paraformaldehyde. By complying with the operating manual, the reaction solution was prepared and added into each well to react with EdU. The nuclei were stained with the DAPI solution. Furthermore, the results were observed and photographed under a fluorescence microscope.

### Cell cycle analysis

MC3T3-E1 cells were seeded into 6-well plates and then cultured with different treatments for 5 days. After trypsin digestion and washing with PBS, the cells were collected and then added to 70% cold ethanol to be fixed for 12 h. Next, propidium iodide (PI) was used to stain the fixed MC3T3-E1 cells. After incubation for 30 min, the samples were analysed using flow cytometry.

### Alkaline phosphatase (ALP) staining and ALP activity assay

MC3T3-E1 cells were seeded into 6-well plates with osteogenesis medium, and then different treatments were applied for 7 days. The samples from the respective groups were divided into two parts: one was used for ALP staining, and the other was used to perform the ALP activity assay. For ALP staining, the samples were fixed with paraformaldehyde and then stained with BCIP/NBT staining solution according to the manufacturer's protocol. The results were observed and then photographed with a camera. To perform the ALP activity assay, the samples were washed and then lysed with lysis buffer, and the OD of lysate was tested with absorbance at 405 nm.

### Alizarin Red S staining and quantification assay

MC3T3-E1 cells were seeded into 6-well plates with osteogenesis medium, and then different treatments were applied for 21 days. Subsequently, the MC3T3-E1 cells were fixed with paraformaldehyde and then incubated with Alizarin Red S solution for 1 h. The results were observed and then photographed with a camera. To perform the quantification assay, the orange–red deposits (calcium nodules) were destained with 10% cetylpyridinium chloride, and then the solution was transferred into 96-well plates. The OD value of the respective well was tested with absorbance at 550 nm.

### Scratch assay

MC3T3-E1 cells were seeded into 6-well plates and then received different treatments for 5 days. Then, the treatment conditions were removed, and fresh normal medium without FBS was added to the plates. A 200-μL pipette tip was adopted to make an artificial wound on the bottom of the plate. The detached cells were washed with PBS and then cultured for 24 h to conduct scratch healing. Cell migration was observed, and the wound was photographed. In addition, the area of the wound and the repaired region were measured with ImageJ software.

### Transwell assay

MC3T3-E1 cells were seeded into 6-well plates and then cultured with different treatments for 5 days. Subsequently, 1 × 10^5^ cells from different groups were collected in 200 μL medium without FBS and then added to the upper chamber. Moreover, 800 μL medium with 20% FBS was added to the lower chamber. After 24 h, cells on the upper surface were wiped away, and those on the lower surface were stained with 0.1% crystal violet. Photomicrographs were taken, and the migrated cells were counted in three random microscope fields.

### Western blot

After different treatments were performed for 5 days, MC3T3-E1 cells were collected and then lysed with RIPA buffer to extract total protein. The total protein concentration was examined with a BCA protein assay. After premixing with loading buffer, equal total protein amounts (20 μg) were separated by 10% SDS-PAGE and then transferred to PVDF membranes. After the protein was incubated overnight with the primary antibody, the HRP-labelled secondary antibody was used to bind to the primary antibody. An enhanced chemiluminescence kit was adopted to detect the antigen-antibody complex.

### Animal models and treatment

The animal experiments were performed under the approval and supervision of the Animal Ethics Committee of First Hospital of Jilin University (Approval No. 2020-010). Thirty male SD rats were purchased from Charles River Laboratories (Beijing, China) and maintained in the Experimental Animal Centre of Translational Medicine in the First Hospital of Jilin University. All rats were kept in caged rooms at 22–24 °C, 55% humidity and a 12 h light/dark cycle. Water and chow were in good supply.

After being adaptively fed for one week, 30 male rats randomly fell to 3 groups, with 10 rats in each group, which included the NC group, the DM group and the LMHFV group. Diabetes was induced in the rats in the DM and LMHFV groups by intraperitoneal injection of STZ (60 mg/kg) dissolved in sodium citrate solution. The rats in the NC group were injected with equal volumes of sodium citrate solution. After three days, blood samples were collected from the rats and then tested with a Roche glucose metre. Rats with blood glucose concentrations ≥ 16.7 mmol/L were considered diabetic rats. After one week of induction of diabetes, LMHFV treatment was applied to the rats in the LMHFV group through an LMHFV platform, and the LMHFVs were 35 Hz, 0.25 g, 15 min/day and 5 days/week, as previously reported by the authors [16]. Plasma glucose and body weight of each rat were measured at the 4th week and the 8th week.

The intervention of the LMHFV lasted for 8 weeks. All rats were sacrificed by CO_2_ asphyxiation. The femurs of rats were obtained, and the soft tissues of the femurs were dissected away. The left femurs were wrapped in gauze with wet salt water and then preserved at 4 °C. The right femurs were preserved with paraformaldehyde.

### Measurements of bone morphometry and bone microarchitecture

Photography was performed on right femurs. The length and width of femurs were measured with a Vernier calliper. The femoral length was defined as the distance from the femoral head to the distal condyle, and femoral width was defined as the distance from the medial surface to the lateral surface at the midshaft.

To analyse the microarchitecture of trabecular bone in the femur, a micro-CT system (Bruker MicroCT, Belgium) was used to scan the trabecular image. Right femurs were fixed in tubes and then scanned at 70 kV and 100 mA. The 2.0-mm region of interest (ROI) in the trabeculae was obtained below the growth plate in the distal metaphysis. Commercial software (e.g. CT dataviewer, CTAn and CTvol) was used to quantitatively analyse the ROIs. The bone mineral density (BMD), bone volume/tissue volume (BV/TV), trabecular thickness (Tb. Th), trabecular number (Tb. N) and trabecular separation (Tb. Sp) of the ROI were ascertained, and 3D reconstruction of the ROI was performed.

### Measurements of bone mechanical properties

To examine the mechanical properties of femurs, the three-point bending test was performed in the left femur with a universal material test machine (AG-X plus, Japan). The left femurs were rewarmed for 1 h and then placed horizontally on the fixture with a 20 mm span of the test machine. Compression at a speed of 1 mm/min was applied in the middle of the whole femur sham. Materials Testing Software (TRAPEZIUMX-V, Japan) was adopted to record the mechanical signal in real time and generate a load-deformation curve. The ultimate load, stiffness and energy absorption of femurs were obtained from the load-deformation curve.

### Measurements of bone matrix composition

After the three-point bending test, the broken left femurs were collected. A segment of cortical bone of the femur along the horizontal plane was excised. After alcohol dehydration, the embedding process in epoxy resin and grinding, the samples for Raman spectroscopy (RS) were obtained. A Raman microscope (Horiba LabRAM, France) with a 785-nm wavelength laser source was adopted to obtain the spectra of femur cortical tissue. The laser was focused on a cross section of the femur cortex with a 50× magnification objective. The average of spectra obtained from three different sites of the femur cortex were employed to reflect the composition of the bone matrix. Curve fitting of Raman spectra was performed with Origin analysis software (OriginLab, USA), and the band area was calculated. The definition and calculation of the main Raman spectra-based compositional parameters in this study are described below: mineral-to-matrix ratio (area of v_1_PO_4_^3−^ [960 cm^−1^] band: area of Amide I [1660 cm^−1^] band), carbonate to phosphate ratio (area of v_1_CO_3_^2−^ [1060 cm^−1^] band: area of v_1_PO_4_^3−^ [960 cm^−1^] band), mineral crystallinity (inverse of v_1_PO_4_^3−^ [960 cm^−1^] band width at half-maximum intensity).

### Statistical analysis

The data from three repeated experiments are expressed as the mean and standard deviation. Comparisons among different groups were drawn by one-way ANOVA followed by Bonferroni post-test with SPSS 26.0 software (Chicago, USA). *P* values below 0.05 were considered statistically significant.

## Results

### LMHFV alleviated the inhibitory effect of high glucose on the proliferation of MC3T3-E1 cells

**Fig. 1:**
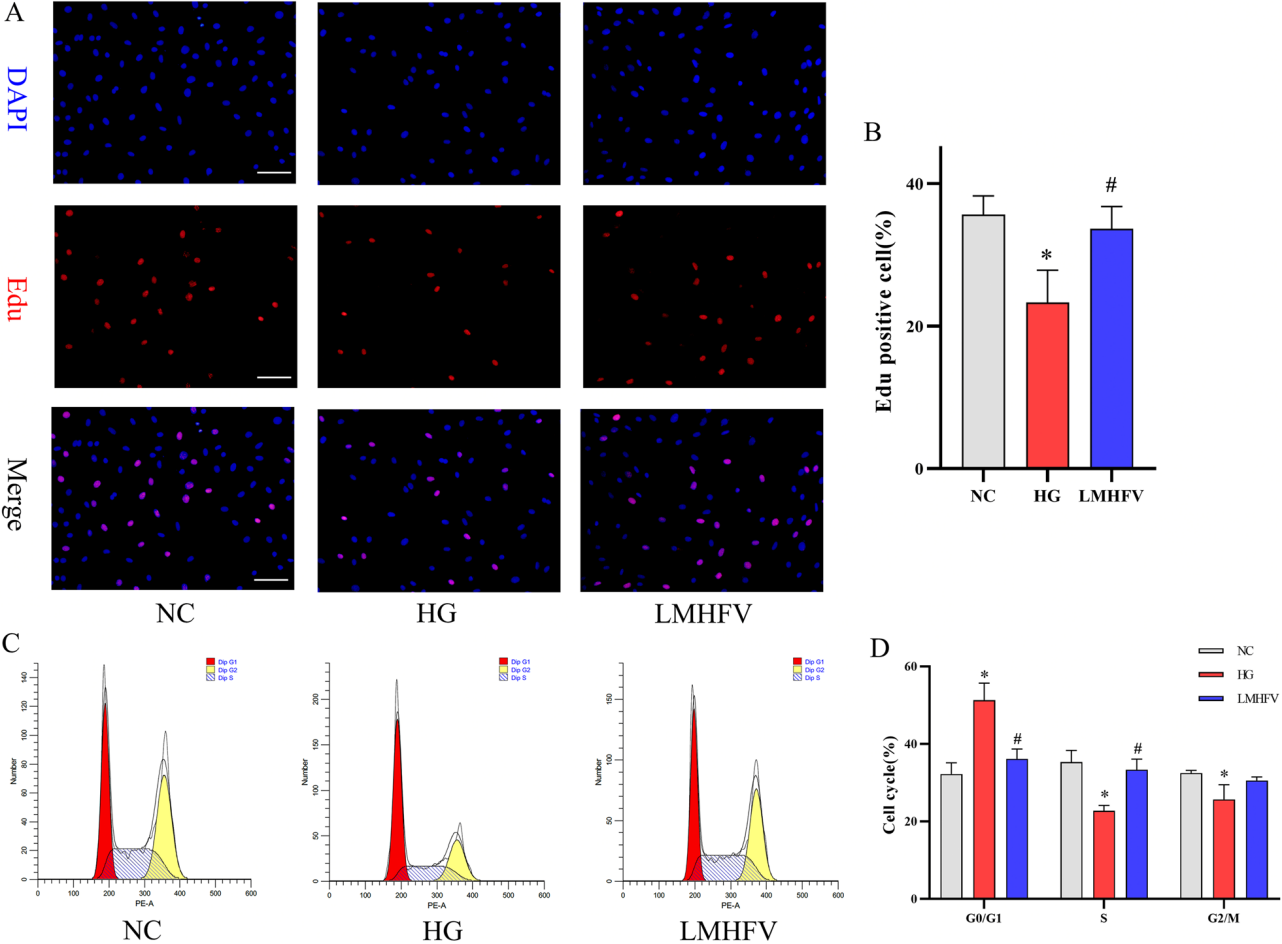
Effects of LMHFV on the proliferation and cell cycle of HG-induced MC3T3-E1 cells. **A** EdU staining was performed to detect the cell proliferation of each group, and the fluorescence image of EdU-positive cells reflected the proliferation of each group. Scale bar = 100 μm. **B** The percentage of EdU-positive cells in each group was calculated, and the results are presented in the bar graph. **C** The percentages of cells in G0/G1, S and G2/M phase are shown in the bar graph. **D** DNA content-based (stained with PI) cell cycle analysis was conducted, and the cell cycle variations of cells from the respective groups, including G0/G1, S and G2/M, were exhibited using flow cytometry histograms. *P<0.05, compared with the NC group. ^#^*P*<0.05, compared with the HG group.

First, an EdU assay was performed to examine the proliferation of MC3T3-E1 cells under different experimental conditions. Variations in the proportion of EdU-positive cells reflected cell proliferation under different treatment conditions. According to Fig. 1A, B, the proliferation of MC3T3-E1 cells was significantly reduced after high glucose treatment for five days, while the application of LMHFV increased the proliferation of MC3T3-E1 cells against high-glucose treatment. Since variations in cell proliferation may be attributed to variations in the cell cycle, this study further investigated the cell cycle changes of MC3T3-E1 cells under different treatments using flow cytometry. As shown in Fig. 1C, D, the cell cycle analysis by flow cytometry revealed the occurrence of G0/G1 arrest in MC3T3-E1 cells after the cells were exposed to high glucose. The application of LMHFV inhibited G0/G1 arrest attributed to high glucose and increased the ratio of cells in S phase, which demonstrated the acceleration of DNA synthesis and tendency for proliferation.

### LMHFV promoted the differentiation and mineralization of MC3T3-E1 cells under high-glucose conditions

**Fig. 2:**
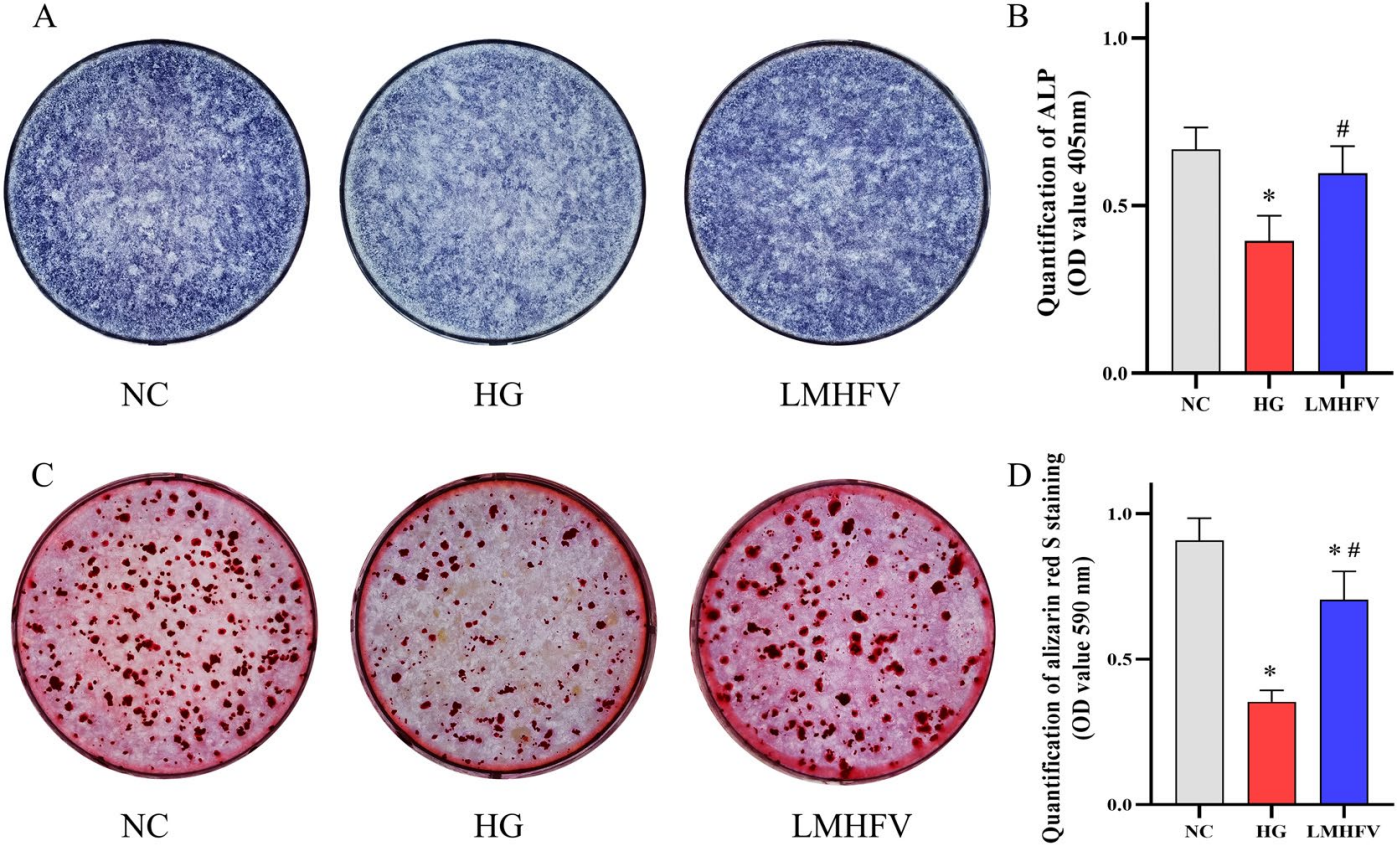
Effects of LMHFV on the differentiation and mineralization of HG-induced MC3T3-E1 cells. **A** BCIP/NBT staining was performed to assess the ALP activity of the respective groups. **B** The ALP activity of each group was quantitatively analysed by using an ALP activity kit. The ALP activity was reflected by the absorbance of lysate at 405 nm, and the results are illustrated in the bar graph. **C** Alizarin Red S staining was performed to assess the mineralization of each group. **D** Mineralization of each group was quantitatively analysed by using cetylpyridinium chloride to resolve calcium nodules, and mineralization was reflected by the absorbance of lysate at 550 nm, with the results illustrated in the bar graph. *P<0.05, compared with the NC group. ^#^*P*<0.05, compared with the HG group.

Preosteoblasts differentiate into mature osteoblasts after being activated by a variety of factors, and they facilitate new bone formation through mineralization. Thus, after different treatments in MC3T3-E1 cells, alkaline phosphatase (ALP) staining and ALP activity assays were adopted to test the differentiation, while Alizarin Red staining and quantification assays were performed to test the mineralization. According to Fig. 2A, B, the intensity of the blue–purple colour revealed the activity of ALP. As demonstrated by the lighter blue–purple colour, exposure to high glucose decreased the activity of ALP in MC3T3-E1 cells. In addition, the darker blue–purple colour indicated that the application of LMHFV alleviated the decrease in the activity of ALP upon exposure to high glucose. Moreover, the quantification of ALP activity was examined, and the results correlated with the staining. Subsequently, Alizarin Red staining and quantification assays were performed to investigate the mineralization of MC3T3-E1 cells in different treatments. The area of orange–red deposits (calcium nodules) reflected the magnitude of the mineralization. As shown in Fig. 2C, D, fewer areas with orange–red deposits indicated HG-induced suppression of mineralization in MC3T3-E1 cells. However, the inhibition of mineralization by high glucose was attenuated after the application of LMHFV: the area with orange–red deposits increased. Furthermore, the orange–red deposits were quantified, and the results were consistent with staining, which demonstrated that LMHFV could alleviate the inhibition of mineralization in HG-induced MC3T3-E1 cells.

### LMHFV improved the migration ability of osteoblasts damaged by high glucose

**Fig. 3:**
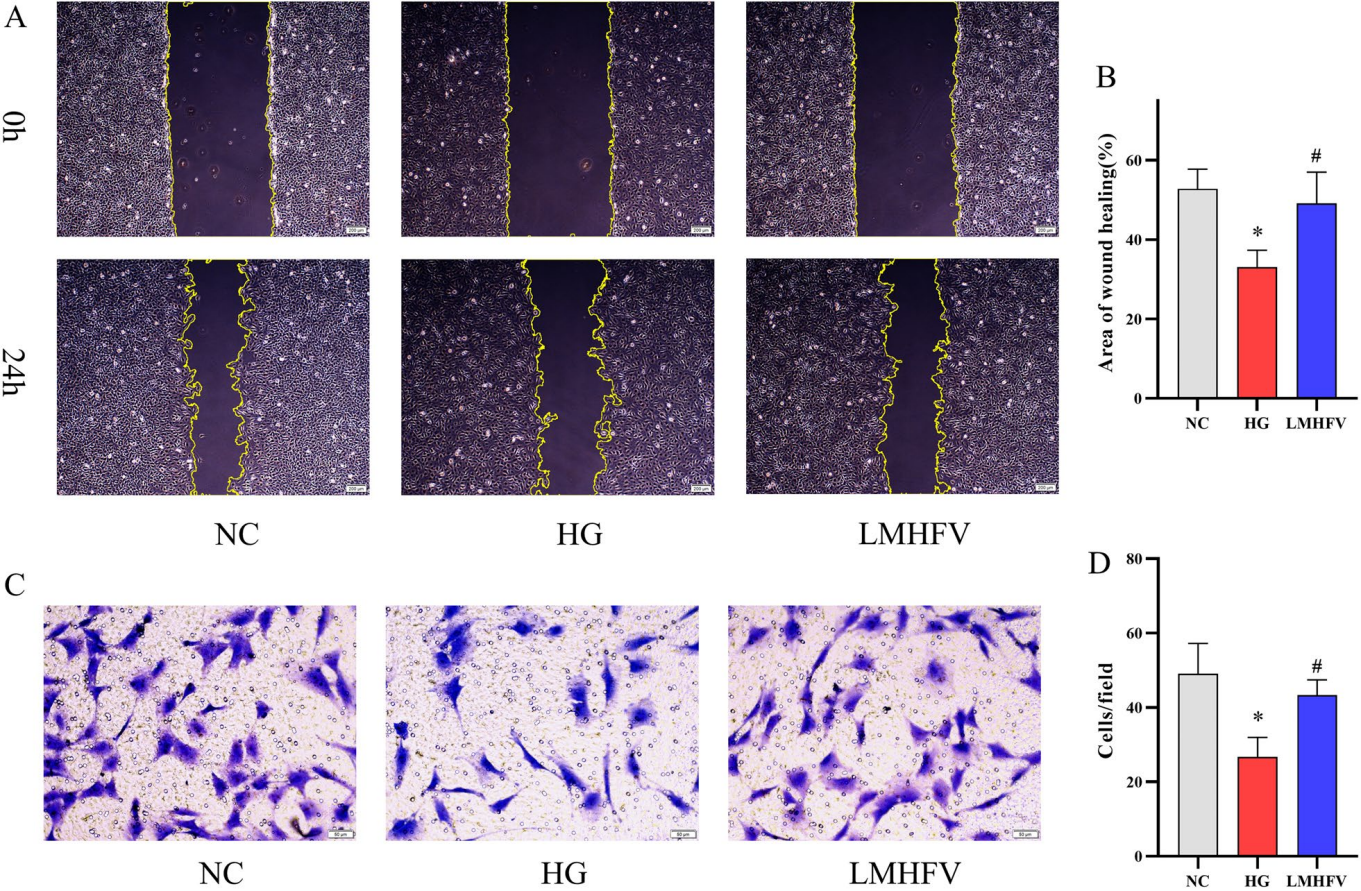
Effects of LMHFV on the migration ability of HG-induced MC3T3-E1 cells. **A** A scratch assay was performed to investigate the cell migration of each group. Phase-contrast images of MC3T3-E1 cells at 0 h and 24 h after the wound scratch were recorded. **B** The percentage of the wound healing area was calculated for different groups, with the results illustrated in the bar graph. **C** Transwell assays were performed, and cells successfully passing through the chamber were stained and then photographed. **D** The cell number was calculated, and the results are illustrated in the bar graph. *P<0.05, compared with the NC group. ^#^*P*<0.05, compared with the HG group.

Osteoblasts still exhibit migration ability when cultured in vitro. A scratch assay based on a wound healing model was used to identify the migration and movement characteristics of cells in vitro. To investigate the effect of LMHFV on MC3T3-E1 cells under high glucose, a scratch assay was performed, and the area of wound closure from different groups was measured and then compared. As shown in Fig. 3A, B, the migration behaviour of MC3T3-E1 cells was inhibited by high-glucose conditions. LMHFV showed the ability to improve the migration ability of MC3T3-E1 cells affected by high glucose. To test this further, a Transwell assay with cells moving from the upper chamber to the lower chamber and reflecting the migration ability of cells was performed. According to Fig. 3C, D, LMHFV application improved the decrease in the number of cells successfully passing through the chamber under high glucose. Overall, LMHFV improved the migration ability of MC3T3-E1 cells inhibited by high glucose.

### LMHFV played a beneficial role in HG-induced MC3T3-E1 cells through the GSK3β/β-catenin pathway

**Fig. 4:**
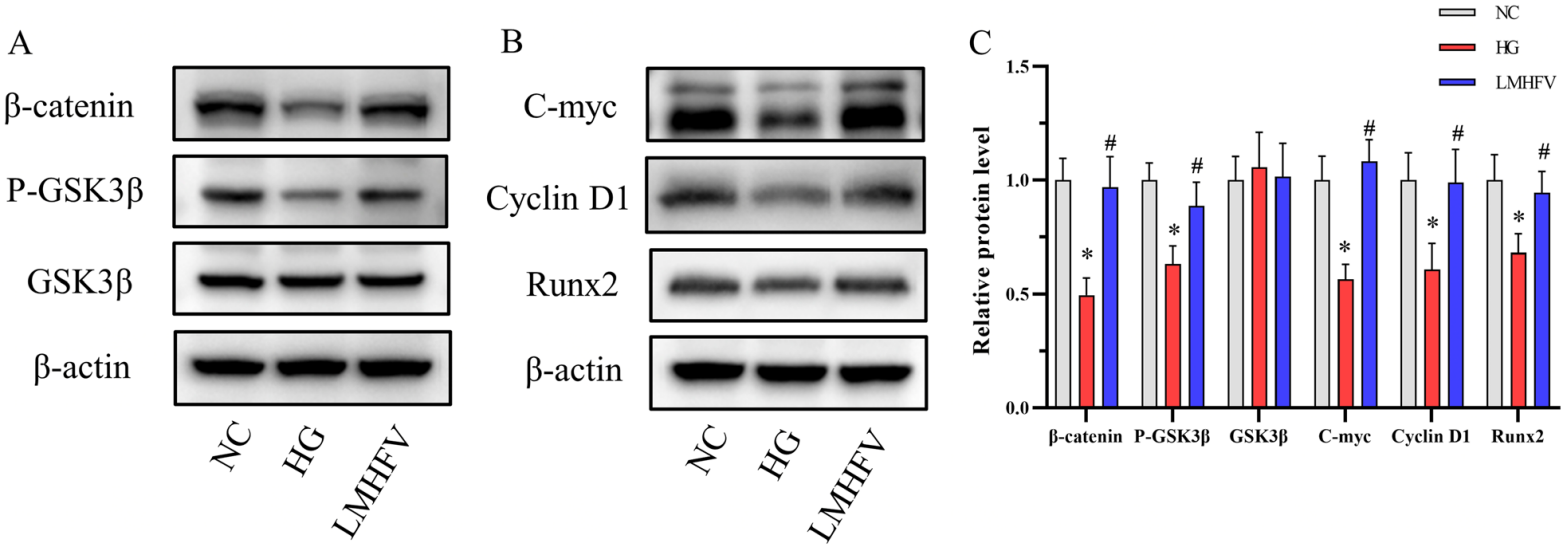
Effects of LMHFV on the GSK3β/β-catenin pathway and related protein expression in HG-induced MC3T3-E1 cells. **A**, **B** Western blotting assays were performed to detect the protein expression of GSK3β, p-GSK3β, β-catenin, C-myc, Cyclin D1 and Runx2. The results are presented as the western blotting bands. **C** The grey value of the western blot band of each group was calculated, and the relative protein expression was compared, as presented in the bar graphs. *P<0.05, compared with the NC group. ^#^*P*<0.05, compared with the HG group.

According to previous experimental results, LMHFV exhibited a protective effect against high glucose with respect to the proliferation, differentiation and migration of MC3T3-E1 cells. This study further investigated the molecular mechanism of the protective effect of LMHFV in HG-induced MC3T3-E1 cells. To determine whether the GSK3β/β-catenin signalling pathway was involved in MC3T3-E1 cells after different treatments, the protein expression levels of GSK3β, p-GSK3β and β-catenin were first assessed using western blotting. According to Fig. 4A, C, no significant change was found in the GSK3β expression level observed under different conditions. However, the p-GSK3β and β-catenin expression levels were inhibited by high glucose, while the application of LMHFV alleviated the inhibition induced by high glucose.

Next, a western blotting assay was performed again to examine the protein expression levels of C-myc, Cyclin D1 and Runx2, which are considered protein markers associated with the proliferation, cell cycle and differentiation of osteoblasts. According to Fig. 4B, C, high glucose treatment decreased the C-myc, Cyclin D1 and Runx2 expression levels, while the application of LMHFV alleviated their inhibition by high glucose in MC3T3-E1 cells. The GSK3β/β-catenin inhibitor XAV939 was used to further elucidate the role of the GSK3β/β-catenin pathway in the LMHFV effect. As shown in Additional file 1: Fig. 1A, B and C, the activation of the GSK3β/β-catenin pathway by LMHFV was inhibited after the use of inhibitors. Furthermore, the elevated C-myc, Cyclin D1 and Runx2 levels due to LMHFV application were also reduced by inhibiting the GSK3β/β-catenin pathway.

### LMHFV enhanced body weight, reduced blood glucose and increased femur length and width in diabetic rats

**Fig. 5:**
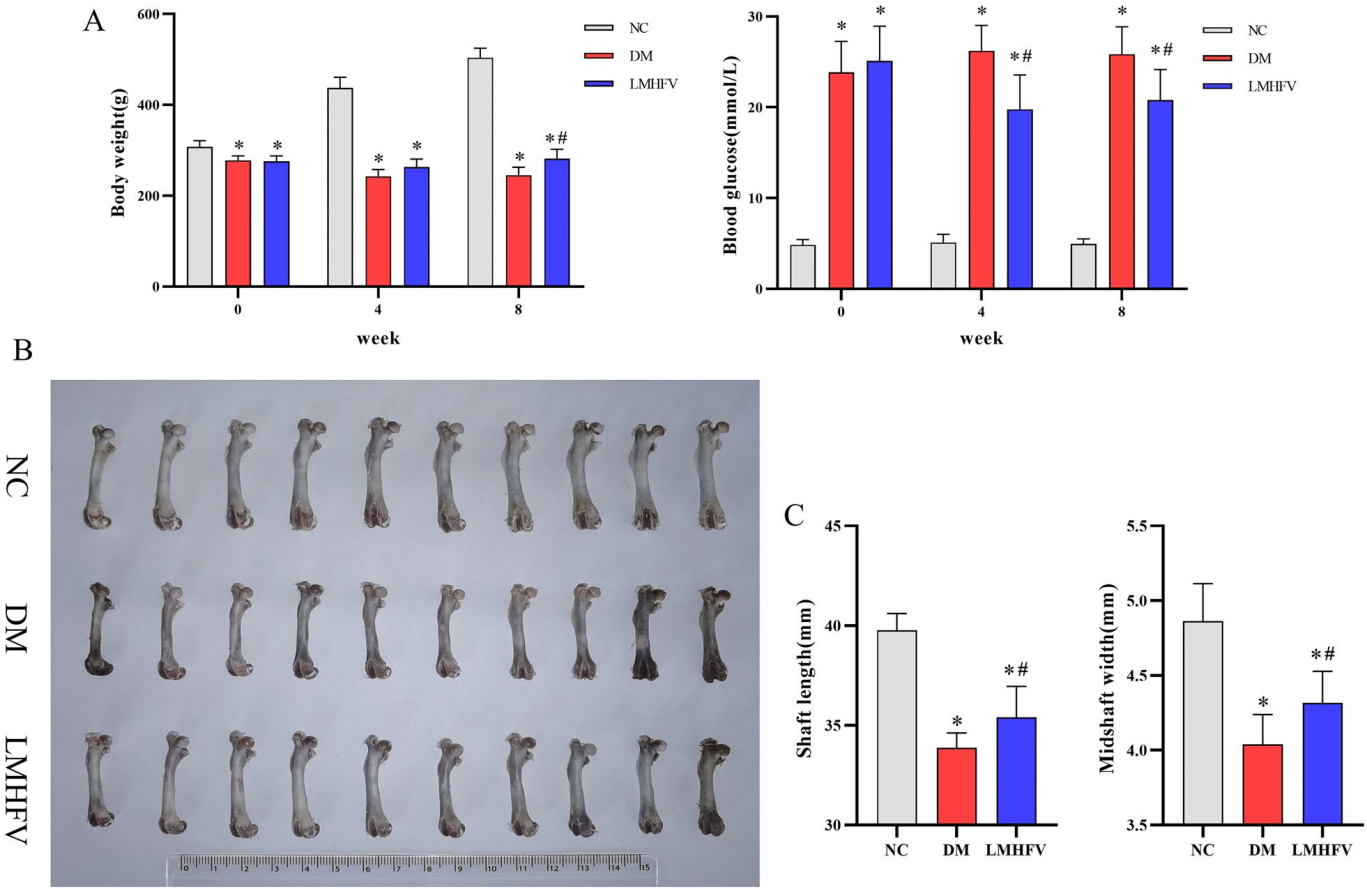
Effects of LMHFV on the body weight, blood glucose and bone morphometry of diabetic rats. **A** Variations in body weights and blood glucose of rats from different groups at the end of the fourth and eighth weeks. **B** General right femur images of rats from different groups. **C** Bar graphs present the measured length and width of the right femurs. *P<0.05, compared with the NC group. ^#^*P*<0.05, compared with the DM group.

As shown in Fig. 5A, compared with the rats of the NC group, STZ injection resulted in a significant increase in blood glucose and weight loss in rats of the DM and LMHFV groups, which were the typical manifestations of type 1 diabetes. For the comparison between the DM group and the LMHFV group, a significant decrease in blood glucose in the fourth week and the eighth week was observed, and the weight increase occurred during the eighth week. Furthermore, the right femur of rats (including the femur length and width) was measured. As indicated in Fig. 5B, C, the length and width of the femurs in the DM group were the shortest. Even though they were still lower than those in the NC group, LMHFV treatment was capable of increasing the length and width of diabetic rat femurs.

### LMHFV increased bone mineral density and improved bone microarchitecture in diabetic rats

**Fig. 6:**
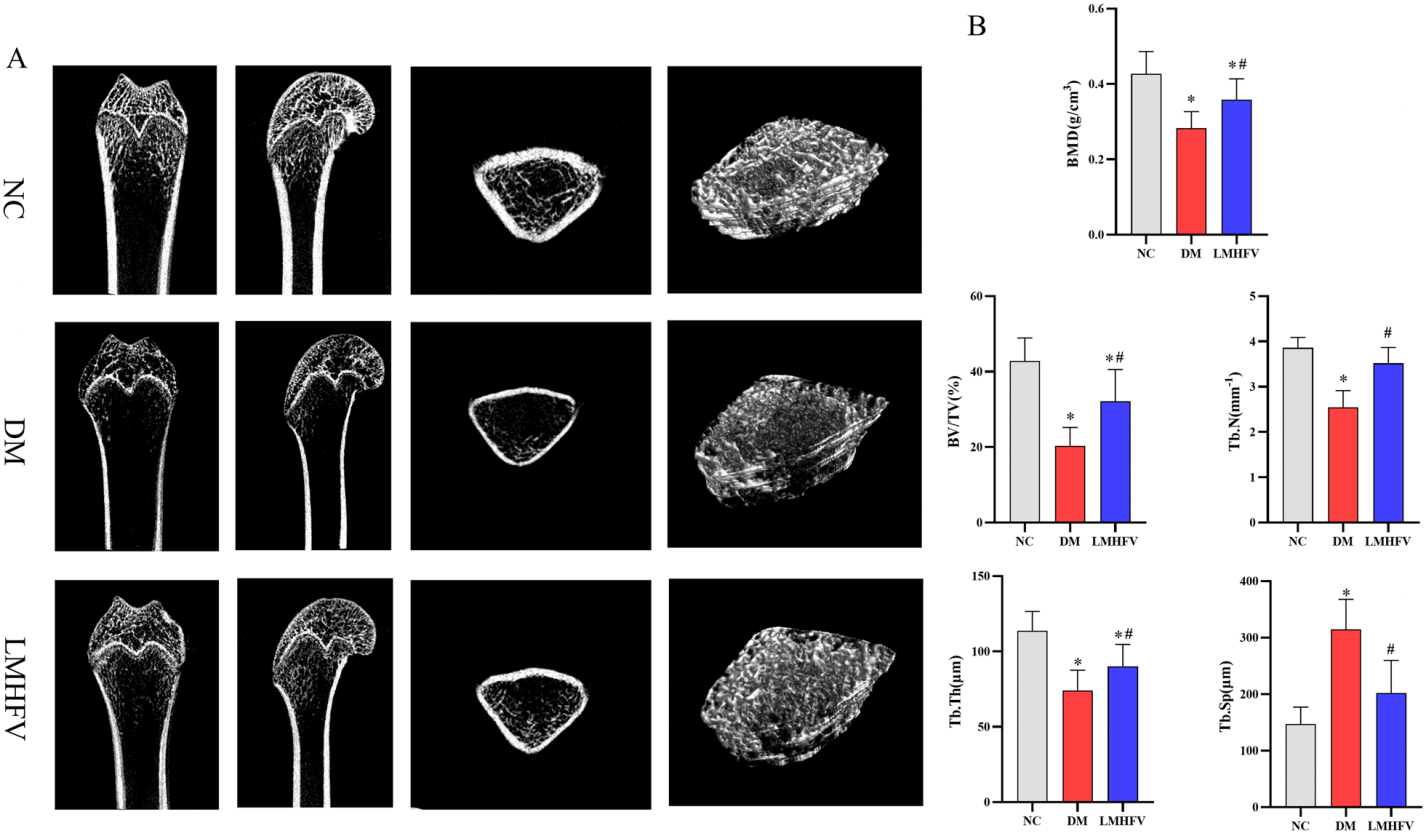
Effects of LMHFV on the femur microstructure of diabetic rats. **A** Typical microCT images of coronal, sagittal and transverse sections of trabecular bone are presented. Images of the 3D reconstruction performed for the region of interest (ROI) are presented. **B** Trabecular parameters (e.g. BMD, BV/TV, Tb. Th, Tb. N and Tb. Sp) were analysed, and the results are shown in the bar graphs. *P<0.05, compared with the NC group. ^#^*P*<0.05, compared with the DM group.

Typical microCT images and 3D reconstruction images of rat distal femurs demonstrated a remarkable decrease in bone mass and significant damage to the microstructure of the femur attributed to diabetes, as shown in Fig. 6A. Specifically, diabetes reduces the number of trabecular bones and decreases the quality of trabecular bone in rats. Through quantitative analysis, as shown in Fig. 6B, the results showed that compared with the NC group, the BMD, BV/TV, Tb. Th and Tb. N of trabecular were decreased significantly, while Tb. Sp were increased in the rats of the DM group. LMHFV treatment resulted in an increase in BMD, BV/TV, Tb. Th, and Tb. N, with a decrease in Tb. Sp of the rat femurs in the LMHFV group compared with those of the DM group. Especially with respect to Tb. N and Tb. Sp, the LMHFV eliminated the effect of DM in the femur since no significant variations were observed between the NC group and the LMHFV group.

### LMHFV improved the biomechanical properties of femurs in diabetic rats

**Fig. 7:**
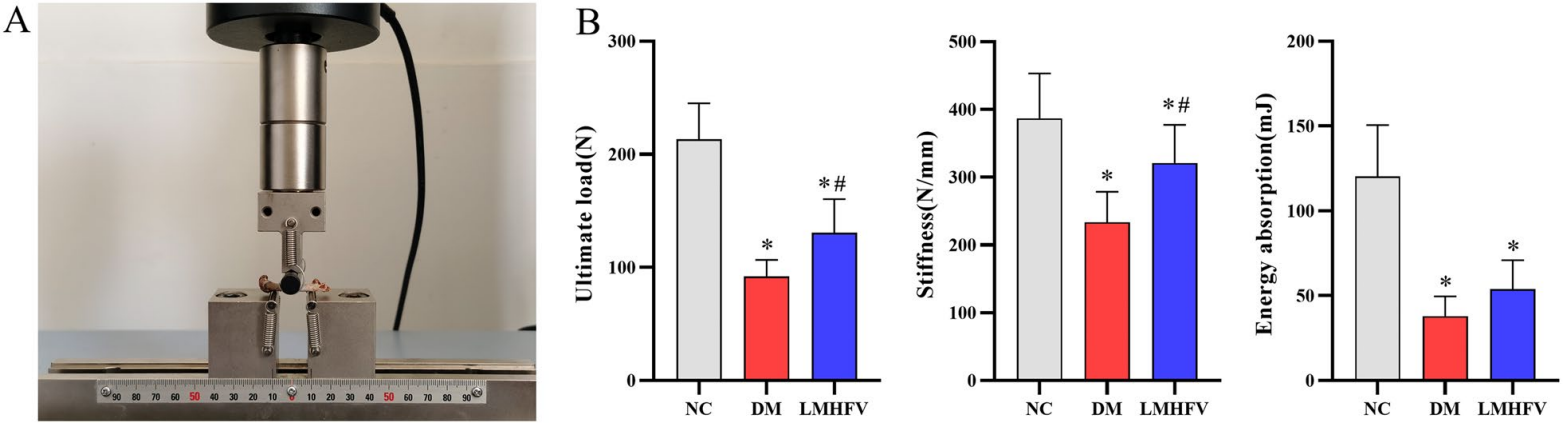
Effects of LMHFV on the biomechanical properties of femurs in diabetic rats. **A** Diagram of three-point bending tests at mid-shaft femur sites. **B** The biomechanical properties of cortical bone (e.g. ultimate load, stiffness and energy absorption) are shown in the bar graphs. *P<0.05, compared with the NC group. ^#^*P*<0.05, compared with the DM group.

Three-point bending tests helped to investigate the variations in the mechanical properties of rat femurs with different treatments. As indicated in Fig. 7A, B, DM significantly reduced the mechanical properties of the femur, and there were different degrees of reduction in the ultimate load, stiffness and energy absorption. When the results from the DM and LMHFV groups were compared, this study indicated that LMHFV could improve the ultimate load and stiffness of the femur, whereas no effect of LMHFV was observed on the energy absorption of the femur.

### LMHFV modified the bone matrix composition of femurs in diabetic rats

**Fig. 8:**
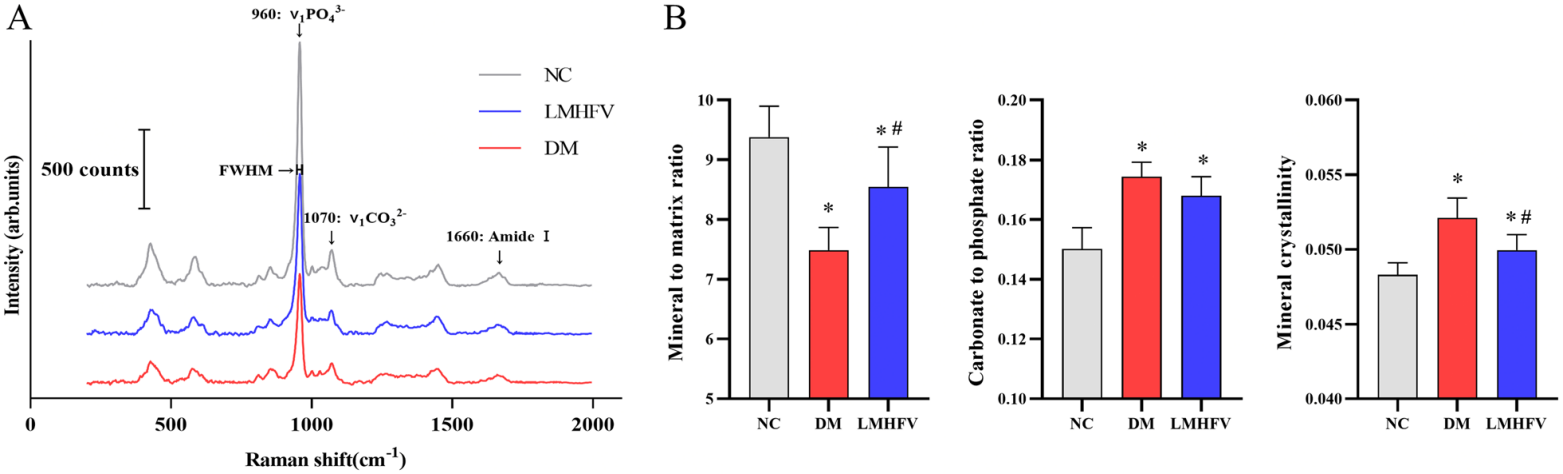
Effects of LMHFV on the bone matrix composition of femurs in diabetic rats. **A** Typical Raman spectra of cortical bone of femur from different groups. The main bands of utility in bone matrix composition analysis for femur are labelled. **B** The mineral to matrix ratio, carbonate to phosphate ratio and mineral crystallinity of femurs from different groups were calculated, with the results shown in the bar graphs. *P<0.05, compared with the NC group. ^#^*P*<0.05, compared with the DM group

Raman spectroscopy was performed to further analyse the variations in femur tissue composition attributed to diabetes or LMHFV treatment. As shown in Fig. 8A, typical Raman spectra of femurs from different groups were exhibited. According to the analysis of the spectral signal, a comparison was drawn for the composition of the femur under different conditions. As shown in Fig. 8B, the decreased mineral to matrix ratio in the DM group was improved with LMHFV treatment. Moreover, the increased mineral crystallinity of the femur attributed to DM was reduced with LMHFV treatment. However, no effect of the LMHFV treatment on the carbonate to phosphate ratio was observed, and the carbonate to phosphate ratio of the femur increased in both the DM and LMHFV groups compared with the NC groups.

## Discussion

Numerous studies have investigated the mechanism of diabetes-induced osteoporosis. It is generally considered that excessive abnormal substances, including extra glucose, advanced glycation end products (AGEs) and inflammatory molecules, accumulate over a long period to impose unfavourable conditions damaging bone cells in diabetes patients [17]. For hyperglycaemia, on the one hand, excessive glucose caused osteocyte death, variations in the microstructure of bone lacuna and bone fragility. On the other hand, hyperglycaemia decreases the number and activity of osteoblasts while increasing the number and activity of osteoclasts, which causes an imbalanced ratio and dysfunction between osteoblasts and osteoclasts, thereby resulting in impaired bone remodelling function and accelerated bone loss [18].

Physical stimuli such as mechanical vibration, shock waves and electromagnetic fields have been proven to promote bone formation to accelerate fracture healing and improve osteoporosis [19, 20]. The results from the in vitro experiments revealed the promotion effect of LMHFV on both osteogenic differentiation and proliferation of osteoblasts [21]. Osteoblasts are considered the main functional cells for bone formation and are responsible for the synthesis, secretion and mineralization of bone matrix, which is accompanied by dynamic variations in osteoblast proliferation, differentiation and migration [22]. We speculated that LMHFV may improve bone loss induced by diabetes by promoting bone formation. The present study provides the first effort to investigate the effect of LMHFV on MC3T3-E1 cells induced by high glucose in vitro. Moreover, the underlying mechanism of the LMHFV effect and feasibility of LMHFV in ameliorating diabetic bone loss in vivo were also demonstrated.

As reported in existing studies, high glucose can significantly inhibit osteoblast viability and proliferation [23], while LMHFV can increase the proliferation of osteoblasts [24]. In this study, after the application of LMHFV to high glucose-induced MC3T3-E1 cells, the inhibition of cell proliferation attributed to high glucose was significantly alleviated. As suggested by the cell cycle analysis results, LMHFV reduced the proportion of MC3T3-E1 cells in G0/G1 phase and increased the proportion of MC3T3-E1 cells in S phase. These results further confirmed the promoting effect of LMHFV on the proliferation of osteoblasts. As reported previously, high glucose could inhibit the expression of alkaline phosphatase and the formation of calcium nodules during the osteogenic differentiation of osteoblasts [25]. Through alkaline phosphatase staining and Alizarin Red staining, this study suggested that LMHFV could alleviate the inhibition of differentiation and mineralization attributed to high glucose in MC3T3-E1 cells. During bone remodelling, preosteoblasts resting in the stem cell pool started to migrate to the bone formation locus [26]. The migration potential of MC3T3-E1 cells was reported to be inhibited by high glucose [27]. A previous study reported that the recruitment of mesenchymal stem cells could be enhanced by LMHFV [28]. Thus, this study conjectured that LMHFV could improve the migration ability of osteoblasts damaged by high glucose, and the results from wound healing and Transwell assays confirmed this conjecture.

Since we reported that LMHFV could alleviate the inhibitory effect of high glucose on the proliferation, differentiation and migration of MC3T3-E1 cells, the underlying molecular mechanism of the LMHFV effect in HG-induced MC3T3-E1 cells was further investigated. The GSK3β/β-catenin pathway has been considered to be involved in the regulation of proliferation, differentiation and migration of osteoblasts [29]. Under the regulation of upstream signals, GSK3β is phosphorylated or dephosphorylated. When the Ser9 site of GSK3β was phosphorylated, the activity of GSK3β decreased, and the concentration of β-catenin in the cytoplasm increased due to the decrease in degradation. After β-catenin accumulates in the nucleus, it can cooperate with the transcription factor TCF/LEF to jointly regulate the transcription of downstream target genes to regulate the proliferation and differentiation of osteoblasts [30]. High glucose was found to increase the degradation of β-catenin by inhibiting the phosphorylation of GSK3β, reduce the content of β-catenin in the nucleus and ultimately to cause bone loss [31]. LMHFV was reported to upregulate the expression of β-catenin in bone marrow mesenchymal stem cells and to enhance the expression of Runx2 and Cyclin D1 in MC3T3-E1 cells [32]. Accordingly, the levels of GSK3β, p-GSK3β, β-catenin and downstream targets, including C-myc, Cyclin D1 and Runx2, were detected. Combining the results with or without the GSK3β/β-catenin inhibitor XAV939, we demonstrated that LMHFV alleviated the inhibitory effect of high glucose on the GSK3β/β-catenin pathway to regulate the proliferation, differentiation and migration of MC3T3-E1 cells.

Subsequently, the effect of LMHFV on diabetes-induced osteoporosis in vivo was investigated. By inducing diabetes in rats by STZ, the diabetic rats showed significantly elevated blood glucose levels and weight loss as the typical manifestations of type I diabetes mellitus [33]. With LMHFV treatment, significantly reduced blood glucose was observed, which was consistent with the results reported from previous research in STZ-diabetic rats and db/db mice [34, 35]. However, previous studies suggested that LMHFV did not exert any weight gain effect on STZ-diabetic rabbits [36]. The reasons for the differences might be attributed to different animal models of diseases. Rabbits generally weigh more than rats, and the same period of LMHFV may exhibit a stronger effect in rats than rabbits. The morphological and microstructural character variations of the femur were examined. The measurement of the femur indicated that hyperglycaemia-induced bone showed a decrease in length and width in this study, which was consistent with previous studies [37, 38]. Augmented length and width of the femur in the LMHFV group showed the protective effect of LMHFV in bone against diabetes. The microCT results revealed the microstructure variations of trabeculae at the distal femur. Deterioration in trabeculae is considered a sign of bone loss in diabetes, and sparse and thinned trabeculae are common features [39]. In previous reports, LMHFV was reported to ameliorate trabecular bone deterioration attributed to oestrogen deficiency and promote fracture healing in ovariectomy-induced rats [40]. In our study, we reported that the density of cancellous bone of the femur was increased and that the quantity and quality of trabeculae improved after LMHFV treatment. These results suggested that LMHFV could reduce bone loss and facilitate bone formation.

Degradation in the biomechanical behaviour of bone is closely associated with an increased risk of fracture in diabetes [41]. LMHFV was reported to increase the flexural rigidity of the callus in a fracture healing model of ovariectomized mice [42, 43]. Thus, based on the improvement of bone microstructure by LMHFV, the biomechanical properties of the femur were also examined. Consistent with a previous study, samples from the DM group exhibited lower ultimate load, stiffness and energy absorption of cells in the mechanics test [44]. With the exception of energy absorption, both the maximum load and stiffness of the femur were improved after LMHFV treatment. The bone mechanical properties in normal or disease cases could be associated with bone tissue composition [45]. Alterations in the mineral and matrix composition of femurs in diabetic rats were reported to be associated with mechanical strength changes, and one marked change referred to a reduction in the mineral to matrix ratio [46]. In addition, a significant decline in the bone mineral content and the mineral to matrix ratio was reported in diabetes patients [47]. Thus, the effect of LMHFV on bone composition was evaluated by Raman spectral examination. Decreases in the mineral to matrix ratio and increases in the carbonate to phosphate ratio and mineral crystallinity were identified, which were consistent with those of a previous report [48]. An increase in the mineral-to-matrix ratio of the femur was reported after diabetes was treated with sitagliptin, and the ultimate stress and stiffness were ultimately improved [49]. Thus, due to the recovery in the mineral-to-matrix ratio of femurs in DM rats treated with LMHFV in this study, the improvement of mechanical properties by LMHFV might be associated with the change in bone tissue composition.

## Conclusion

In brief, this study demonstrated that LMHFV alleviated the inhibitory effect of high glucose on the proliferation, differentiation and migration of osteoblasts. The GSk3β/β-catenin pathway was involved in the improvement effect of LMHFV against high glucose. Bone microstructural damage and bone fragility due to diabetes were improved by LMHFV in vivo, and bone tissue composition was also adjusted by LMHFV, which demonstrated that LMHFV might be promising in treating diabetic bone loss.

## Supplementary Information

**Additional file 1: Figure 1.** The impact of XAV939 on the effects of LMHFV on the GSK3β/β-catenin pathway and related protein expression in HG-induced MC3T3-E1 cells. (A. B) Western blotting assays were performed to detect the protein expression of GSK3β, p-GSK3β, β-catenin, C-myc, Cyclin D1 and Runx2. The results are presented as the western blotting bands. (C) The grey value of the western blot band of each group was calculated, and the relative protein expression was compared, as presented in the bar graphs. *P < 0.05, compared with the HG group. #P < 0.05, compared with the HG+LMHFV group. △P < 0.05, compared with the HG+XAV939 group.

## Data Availability

All data generated or analysed during this study have been included in the article.

## Abbreviations

LMHFV: Low-magnitude high-frequency vibration
STZ: Streptozotocin
HG: High glucose
ALP: Alkaline phosphatase
DM: Diabetes mellitus
ROI: Region of interest
RS: Raman spectroscopy
BMD: Bone mineral density
BV/TV: Bone volume/tissue volume
Tb. Th: Trabecular thickness
Tb. N: Trabecular number
Tb. Sp: Trabecular separation
