# Supplementary figures and images for: Protective effects of low-magnitude high-frequency vibration on high glucose-induced osteoblast dysfunction and bone loss in diabetic rats
Source: J Orthop Surg Res. 2021 Oct 30;16:650. doi: 10.1186/s13018-021-02803-w (PMC8557505; doi:10.1186/s13018-021-02803-w)

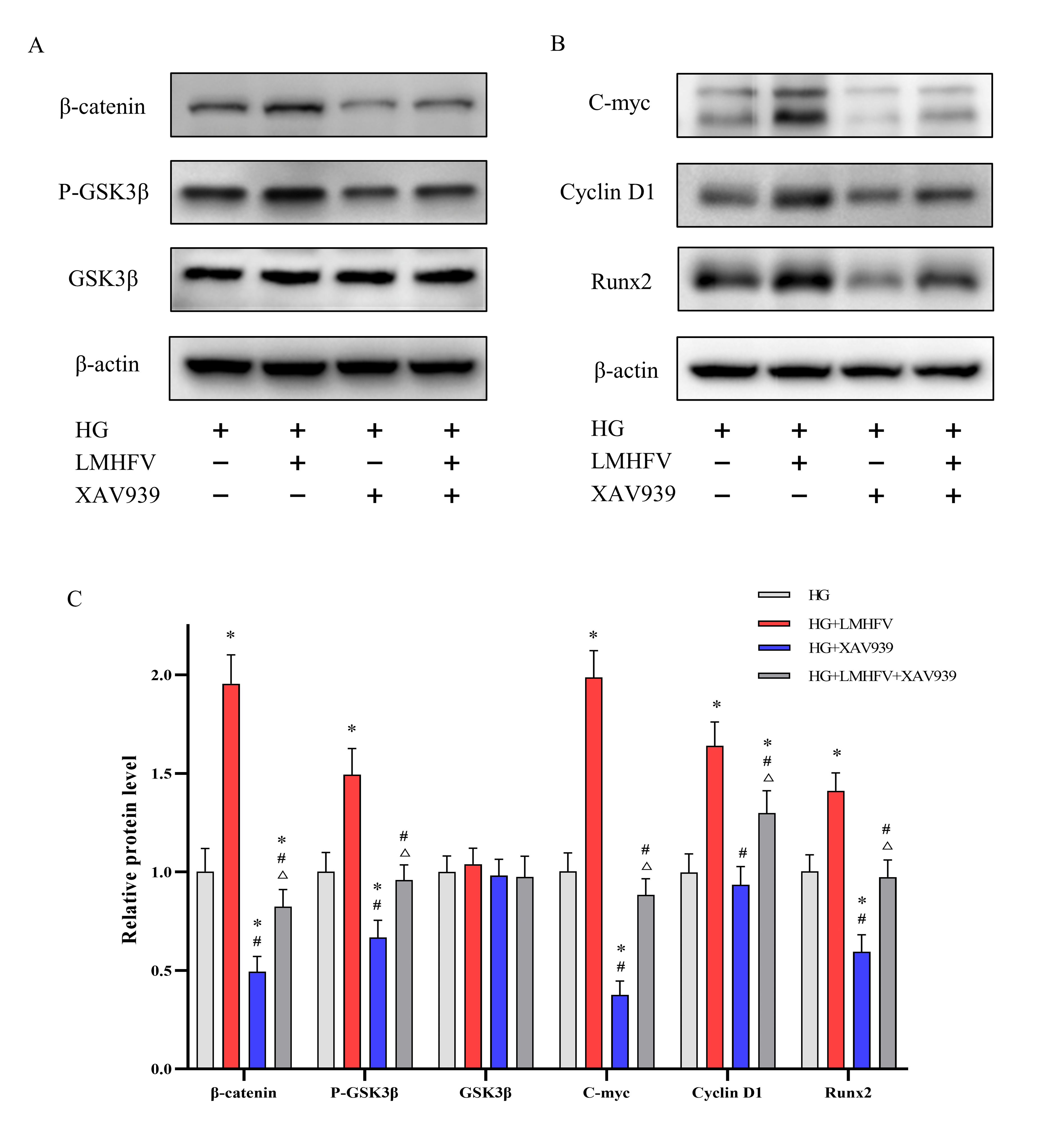

Supplement: Supplementary file 1 — Additional file 1: Figure 1. The impact of XAV939 on the effects of LMHFV on the GSK3β/β-catenin pathway and related protein expression in HG-induced MC3T3-E1 cells. (A. B) Western blotting assays were performed to detect the protein expression of GSK3β, p-GSK3β, β-catenin, C-myc, Cyclin D1 and Runx2. The results are presented as the western blotting bands. (C) The grey value of the western blot band of each group was calculated, and the relative protein expression was compared, as presented in the bar graphs. *P < 0.05, compared with the HG group. #P < 0.05, compared with the HG+LMHFV group. △P < 0.05, compared with the HG+XAV939 group. [file 13018_2021_2803_MOESM1_ESM.png]
